# A randomised controlled trial to evaluate the plaque removal efficacy of sodium bicarbonate dentifrices in a single brushing clinical model

**DOI:** 10.1038/s41405-018-0003-7

**Published:** 2018-06-04

**Authors:** Mary-Lynn Bosma, Kimberly R Milleman, Ivy Akwagyiram, Darren Targett, Jeffery L Milleman

**Affiliations:** 1 GSK Consumer Healthcare, St George’s Avenue, Weybridge, Surrey KT13 0DE UK; 2 Salus Research, 1220-4 Medical Park Drive, Fort Wayne, IN 46825 USA

## Abstract

**Objective:**

To evaluate plaque removal efficacy of dentifrices containing sodium bicarbonate (NaHCO_3_) compared with a non-NaHCO_3_ dentifrice after a single-timed brushing.

**Materials and methods:**

A randomised, controlled, examiner-blinded, four-period, crossover study in 56 adults with a mean whole-mouth plaque index of ≥2.00 (six site modification of Turesky modification of Quigley-Hein Plaque Index [TPI]). Subjects brushed once for one timed minute with a 67% NaHCO_3_ dentifrice with herbs; a 67% NaHCO_3_ dentifrice without herbs; a 62% NaHCO_3_ dentifrice with herbs; or a non-NaHCO_3_ dentifrice without herbs. All contained 923 p.p.m. fluoride as sodium fluoride. Pre- and post-brushing plaque assessments were performed.

**Results:**

Mean TPI score decreased from pre- to post-brushing with all treatments. There were statistically significantly greater reductions in plaque for NaHCO_3_ dentifrices compared to non-NaHCO_3_ (*p* < 0.0001 for all) with no significant differences between NaHCO_3_-containing dentifrices. A post hoc analysis of plaque removal from different oral areas showed statistically significant differences in favour of the NaHCO_3_ dentifrices over the non-NaHCO_3_ dentifrice for almost all surfaces. No adverse events were reported.

**Discussion and conclusion:**

Plaque removal was significantly greater with NaHCO_3_-containing dentifrices compared with a non-NaHCO_3_ dentifrice after a single, timed brushing. There was no effect of herbal tinctures. This study was registered at ClincalTrials.org: NCT03285984.

## Introduction

Dental plaque consists of a number of different proliferating bacterial microcolonies anchored in an extracellular polymeric matrix that coats the teeth.^1,2^ Regular mechanical removal of the plaque biofilm with proper oral hygiene is essential to prevent the development of dental caries, gingivitis and, ultimately, periodontitis.^3,4^

Sodium bicarbonate (NaHCO_3_; baking soda) has been added to many dental products because of its cleansing properties.^5^ Putt et al.^6^ proposed that NaHCO_3_ may decrease plaque levels when brushing either by reducing the viscosity of plaque’s polysaccharide matrix, leading to easier dislodgement, or by plaque displacement by the large NaHCO_3_ crystals. Several studies have shown that NaHCO_3_-containing dentifrices have superior cleaning efficacy to those without NaHCO_3_. For instance, Mankodi et al.^5^ found that a dentifrice containing 65% NaHCO_3_ removed significantly more plaque during a single, timed brushing than two conventional NaHCO_3_-free dentifrices. Putt et al.^6^ showed that dentifrices containing 20–65% NaHCO_3_ exerted a superior and significant cleaning effect following a single, timed brushing compared with both sodium fluoride/silica-based and triclosan/copolymer-based dentifrices. Both of the aforementioned papers identified a potential dose-dependent relationship whereby there appears to be a positive relationship between NaHCO_3_ concentration and enhanced plaque removal. However, it is unclear if the results from these studies are limited to the particular dentifrice formulations tested or whether they can be generalised to all NaHCO_3_-containing dentifrices.

Two of the dentifrices examined here contain a combination of NaHCO_3_ and herbs. The combination of herbs in these dentifrices appear in commercial formulations, such as parodontax® (GSK Consumer Healthcare, Weybridge, UK) and have previously been suggested to have an anti-gingivitis effect owing to bacteriostatic or bacteriocidal properties;^7,8^ however, more recent in vitro studies suggest that when they are incorporated into a dentifrice formulation, any such effects are negligible. For instance, a laboratory study carried out on biofilms comparing the antimicrobial efficacy of a dentifrice with herbs to a triclosan-containing dentifrice demonstrated limited biocidal action.^9^ Recently, an in vitro study showed that NaHCO_3_ could disrupt plaque biofilm via a non-mechanical action,^10^ suggesting that it is this component of these dentifrices that is having a positive action on plaque levels, not the herbs. Nevertheless, herbs are still often included in commercial NaHCO_3_-containing dentifrices to lend the formulation a distinct ‘herbal’ taste.

The objective of this study was to evaluate the plaque removal efficacy of 67 and 62% NaHCO_3_-containing dentifrices, with and without herbs, compared with a marketed dentifrice without NaHCO_3_, in a single, timed brushing clinical model.

## Materials and methods

This randomised, controlled, examiner-blinded, four-period crossover study was conducted at Salus Research Inc., Fort Wayne, IN, USA. Prior to subject recruitment, the study protocol was approved by an independent Institutional Review Board (U.S. IRB Miami, FL 33143; IRB number: U.S.IRB2012SRI/03). The study was conducted in accordance with good clinical practices and the Declaration of Helsinki.^11^ An administrative amendment was made to the protocol that did not affect the study procedures or outcomes. This study was registered at ClincalTrials.org: NCT03285984.

### Subjects

At screening, all potential subjects signed a written informed consent statement then completed a medical history. Healthy subjects aged ≥18 years were eligible for inclusion if they had good dental health based on medical history and oral soft tissue (OST) examination at screening (see ‘exclusion criteria’), with ≥20 gradable, natural, uncrowned teeth with both facial and lingual scorable surfaces. Exclusion criteria included high levels of extrinsic stain or calculus deposits that might interfere with plaque assessments; dental/oral conditions requiring immediate treatment or that could impact the outcome of the study; sensitivity to oral care products (including any of the study materials); severe gingivitis; periodontitis with pocket depth >5 mm affecting more than two teeth; moderate or severe recession; presence of orthodontic bands or appliances, extensive crowns, partial dentures or fixed retainers on the maxillary or mandibular teeth; intra-oral decorative tattoos or tongue/lip piercing; (peri)-oral ulcerations, including herpetic lesions at screening or baseline; participation in another clinical study or receipt of an investigational drug within 30 days of the screening visit; current use of Listerine® mouthwash (Johnson & Johnson Ltd, Wokenham, Berkshire, UK) or any antimicrobial mouthrinse containing chlorhexidine or cetylpyridinium chloride; use of antibiotics within the 2 weeks prior to the first treatment visit or use of any other treatment that would interfere with the study results/conduct. Other exclusion criteria included pregnancy; lactating; type 1 or 2 diabetes mellitus or any medical condition that could put the subject’s health at risk or interfere with the study conduct or results or unwillingness to abstain from smoking on the morning prior to treatment visits or using chewing tobacco over the course of the study.

### Study procedures

At screening, subjects who met eligibility criteria were provided with a 0% NaHCO_3_ washout dentifrice (Colgate® Cavity Protection Dentifrice; Colgate Oral Pharmaceuticals, New York, NY, USA) and toothbrush (Oral-B® 40 Soft Compact Toothbrush; Procter & Gamble Co., Cincinnati, OH, USA) for use for at least 14 days prior to Treatment Visit 1 and at home during the study period. During the study, subjects were not permitted to have their teeth professionally cleaned, have any elective dental procedure or to use any oral hygiene/oral care products (including another toothbrush or dentifrice) other than those administered as part of the study. Chewing gum, interproximal cleaning (using dental floss, water pick or toothpicks) and tongue brushing was also prohibited.

Subjects were scheduled to attend the first of four treatment visits a minimum of 14 days after screening. They were required to abstain from oral hygiene for ~24 h immediately preceding the pre-brushing dental plaque evaluation and to abstain from eating, drinking, smoking, or orally ingesting anything (excluding medications and/or vitamins) for at least 4 h prior to the plaque assessment and until all dental assessments were complete. Subjects were permitted to drink water up until 1 h before the plaque assessment.

At each treatment visit, subjects underwent a full OST examination followed by plaque disclosing with red disclosing solution (GUM RedCote®; Sunstar Americas Inc., Schaumburg, IL, USA). The examiner performed a pre-brushing plaque assessment according to a six site modification of the Turesky modification of the Quigley-Hein Plaque Index (TPI).^12,13^ Each tooth was divided into six areas for scoring: mesiofacial, facial, distofacial, mesiolingual, lingual and distolingual. The TPI was scored as 0 = no plaque; 1 = slight flecks of plaque at the cervical margin of the tooth; 2 = a thin continuous band of plaque (1 mm or smaller) at the cervical margin of the tooth; 3 = a band of plaque wider than 1 mm but covering <1/3 of the crown of the tooth; 4 = plaque covering at least 1/3 but <2/3 of the crown of the tooth or 5 = plaque covering 2/3 or more of the crown of the tooth. The scores from the areas of all teeth were summed and divided by the total number of areas examined to give the mean whole-mouth TPI. Subjects with a mean whole-mouth TPI score ≥2.00 were randomised to study treatment.

This study evaluated the plaque removal efficacy of four non-commercial dentifrices, all of which included 923 ppm fluoride as sodium fluoride (NaF): (i) 67% NaHCO_3_ dentifrice with six herbs (67% NaHCO_3_ + herbs); (ii) 67% NaHCO_3_ dentifrice without herbs (67% NaHCO_3_/no herbs); (iii) 62% NaHCO_3_ dentifrice with six herbs (62% NaHCO_3_ + herbs); (iv) 0% NaHCO_3_ dentifrice without herbs (0% NaHCO_3_/no herbs). NaHCO_3_ concentration was based on that found in commercially available dentifrices.

Subjects were randomised according to a computer-generated schedule provided by the Biostatistics Department of GSK Consumer Healthcare (GSKCH) using a 4 × 4 Williams Square layout that indicated the treatment order. The examiner, study statistician and other employees of GSKCH who may have influenced study outcomes were blinded to treatment allocation. Test products were supplied in plain white tubes to help maintain blinding.

Following the pre-brushing assessment, subjects who met randomisation criteria were instructed to brush their teeth with the assigned dentifrice (1.5 ± 0.05 g) for one, timed, minute under supervision, after which re-disclosing and a post-brushing plaque assessment were carried out. Subjects were allowed to brush with the washout dentifrice following the post-brushing assessment.

A washout period of at least 3 days followed each treatment period, during which subjects were instructed to brush with the washout dentifrice. Subjects completed four treatment visits and brushed once with each of the four dentifrices being assessed in the study. A full OST examination and pre- and post-brushing plaque assessments were performed at each visit. To assess repeatability, on each day that plaque assessments were conducted, the examiner repeated the plaque assessment on two subjects with a minimum of 10 min between repeat plaque assessments of the same subject.

The investigator or site staff documented adverse events (AEs) or abnormalities in the OST examination that occurred from first use of the investigational product (or washout product) until 5 days following last use. The investigator determined whether there was a relationship between the investigational product and the AE, and graded each AE as mild, moderate or severe.

### Statistical methods

Based on a previous study,^6^ it was estimated that a sample size of 50 subjects would provide 90% power (two-sided α of 0.05) to detect a mean treatment difference of 0.15 in the change from baseline TPI score (standard deviation [SD] 0.317, based on a previous GSKCH study [data on file]). A sufficient number of healthy subjects were screened so that a maximum of 56 subjects who fulfilled all the entry criteria could be randomised to ensure that at least 50 evaluable subjects completed all study visits.

The safety population included all randomised subjects who were dispensed any study treatment. The efficacy analysis was performed on the intent-to-treat (ITT) population, defined as all subjects who were randomised to treatment and had at least one post-baseline efficacy measurement. The primary efficacy variable was mean change from baseline in the TPI score (post-brushing score minus pre-brushing score) following a single, timed brushing. This was analysed using an analysis of covariance (ANCOVA) model that included subject as a random effect, treatment group and study period as factors, and two baseline terms as covariates: subject-level baseline score calculated as mean pre-brushing score across all periods within a subject and period level baseline minus subject-level baseline. The primary efficacy analysis compared the mean change from baseline TPI score for the 67% NaHCO_3_ + herbs dentifrice to the 0% NaHCO_3_/no herbs dentifrice. The secondary efficacy analysis compared the mean change from baseline TPI score for all four dentifrices (excluding the primary comparison described above). These treatment comparisons were obtained from the same statistical model specified for the primary efficacy analysis. No multiple comparison adjustments were made as the primary comparison was specified. The assumptions of normality and homogeneity of variance were assessed and upheld. All tests were two-sided and performed at the 5% significance level.

A post hoc exploratory analysis was conducted to investigate the mean change from baseline TPI score in different areas of the mouth defined according to tooth location (posterior, anterior), tooth surface (facial or lingual) and tooth site (body or interproximal). The plaque scores were calculated for the surface and tooth site combinations shown in Table 1 and were analysed using an ANCOVA model as described above. Whole-mouth baseline TPI scores were used as covariates in this model as these provided more information and explained more of the variability than the baseline scores at specific areas of the mouth. Two-sided treatment comparison tests were performed at the 5% significance level.

**Table 1:** Tooth region and surface combinations for post hoc analysis of mean change from baseline in TPI scores for different areas of the mouth

Description	Tooth numbers	Surface	Region	Summary average over
AFB	6–11, 22–27 (12)	F	B	12 × 1 × 1 = 12 sites
AFI			I (D,M)	12 × 1 × 2 = 24 sites
ALB		L	B	12 × 1 × 1 = 12 sites
ALI			I (D,M)	12 × 1 × 2 = 24 sites
PFB	2–5, 12–15, 18–21, 28–31 (16)	F	B	16 × 1 × 1 = 16 sites
PFI			I (D,M)	16 × 1 × 2 = 32 sites
PLB		L	B	16 × 1 × 1 = 16 sites
PLI			I (D,M)	16 × 1 × 2 = 32 sites

*A* Anterior, *P* Posterior, *F* Facial, *L* Lingual, *B* Body, *I* Interproximal, *D* Distal, *M* Mesial

The repeatability of the examiner in conducting plaque assessments was assessed using a weighted kappa coefficient. Repeatability was considered to be excellent if the kappa coefficient was >0.75, fair to good if the kappa coefficient was ≤0.75 to ≥0.4 and poor if the kappa coefficient was <0.4.

## Results

### Subjects

The first subject was enroled in March 2012 with the last subject completing the study in May 2012. All 56 subjects who were screened for the study were randomised to treatment and completed all four study periods. The study population comprised 36 females (64.3%) and 20 males (35.7%). Participant age ranged from 19 to 66 years, with a mean of 44.1 years (SD 11.87). Subjects were White (91.1%) or Black/African American (8.9%).

### Plaque assessment

**Fig. 1:**
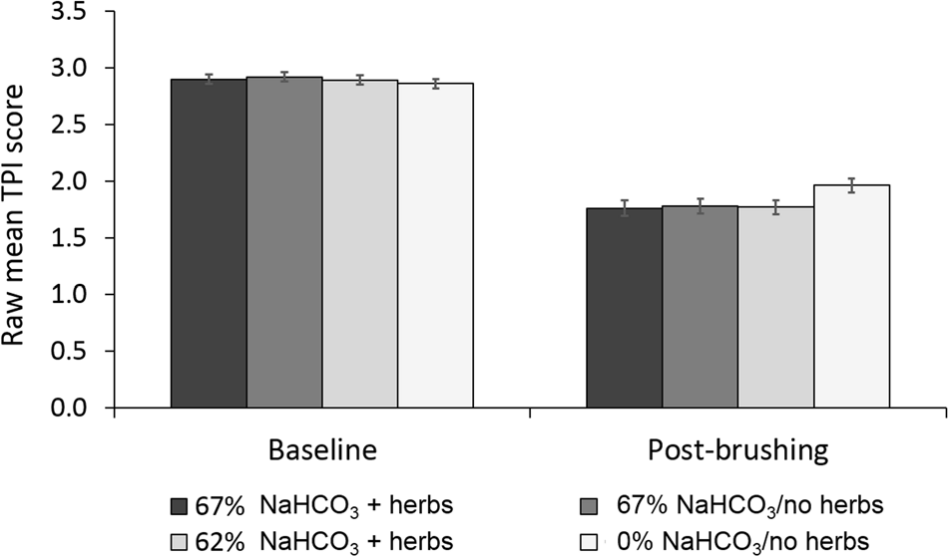
Raw mean whole-mouth TPI score (±SE) at baseline and post-brushing (ITT population). Scores range from 0 (no plaque) to 5 (plaque covering ≥2/3 of the crown of the tooth)

**Fig. 2:**
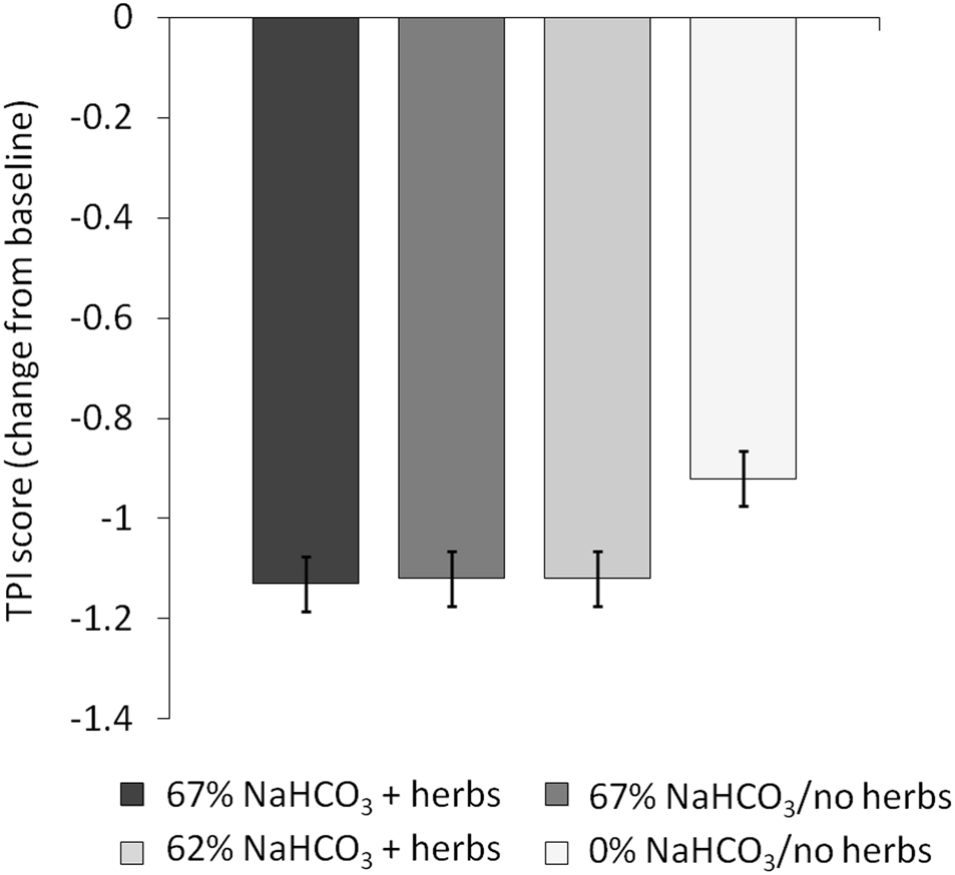
Change from baseline in adjusted mean whole-mouth TPI score (±SE) (ITT population). Scores range from 0 (no plaque) to 5 (plaque covering ≥2/3 of the crown of the tooth)

Mean pre-brushing TPI scores in the 67% NaHCO_3_ + herbs, 67% NaHCO_3_/no herbs, 62% NaHCO_3_ + herbs and 0% NaHCO_3_/no herbs groups were 2.90 (standard error [SE] 0.041), 2.92 (SE 0.042), 2.89 (SE 0.041) and 2.86 (SE 0.041), respectively (Fig. 1). The TPI score decreased from pre-brushing to post-brushing in all treatment groups (Figs. 1, 2). There was a statistically significantly greater reduction in TPI score for the dentifrices containing 67% and 62% NaHCO_3_ compared with the dentifrice containing 0% NaHCO_3_ (*p* < 0.0001 for all comparisons; Table 2). There were no statistically significant differences between any of the NaHCO_3_-containing dentifrices including the 67% NaHCO_3_ dentifices with or without herbs.

**Table 2:** Summary of between-treatment differences in adjusted mean TPI score (ITT population)

Comparison		Difference^a^ (95% CI)	% diff^b^	*p*-value
67% NaHCO_3 _+ herbs	vs 67% NaHCO_3_/no herbs	−0.01 (−0.07, 0.05)	1.3	0.6464
	vs 62% NaHCO_3 _+ herbs	−0.02 (−0.08, 0.04)	1.4	0.6147
	vs 0% NaHCO_3_/no herbs	−0.21 (−0.27, −0.15)	23.1	**<0.0001**
67% NaHCO_3_/no herbs	vs 62% NaHCO_3 _+ herbs	0.00 (−0.06, 0.06)	0.1	0.9653
	vs 0% NaHCO_3_/no herbs	−0.20 (−0.26, −0.14)	21.6	**<0.0001**
62% NaHCO_3_ + herbs	vs 0% NaHCO_3_/no herbs	−0.20 (−0.26, −0.14)	21.4	**<0.0001**

Bolded *p*-values indicate statistical significance

^a^Difference is adjusted mean; a negative difference favours first named treatment

^b^Percent difference: second named treatment taken as reference for percent difference calculation ((difference/reference) × 100%)

The results of the post hoc analysis for plaque removal from different areas of the mouth showed statistically significant differences in favour of the NaHCO_3_-containing dentifrices over the 0% NaHCO_3_ dentifrice for almost all surfaces (Table 3). The largest percent relative plaque removal advantage for the NaHCO_3_-containing dentifrices was found in the anterior lingual interproximal and posterior lingual interproximal areas where ~50% plaque was removed compared to the 0% NaHCO_3_ dentifrice.

**Table 3:** Summary of between-treatment differences in adjusted mean TPI score by tooth location, surface and region (ITT population)

	67% NaHCO_3 _+ herbs vs			67% NaHCO_3_/no herbs vs		62% NaHCO_3_ + herbs vs
	67% NaHCO_3_/no herbs	62% NaHCO_3 _+ herbs	0% NaHCO_3_/no herbs	62% NaHCO_3 _+ herbs	0% NaHCO_3_/no herbs	0% NaHCO_3_/no herbs
Difference^a^ (95% CI) (% difference^b^) *p*-value						
AFB	0.09 (−0.04, 0.23) [−5.0%] 0.1811	0.08 (−0.06, 0.21) [−4.3%] 0.2488	−0.12 (−0.25, 0.02) [7.3%] 0.0837	−0.01 (−0.15, 0.12) [0.7%] 0.8509	−0.21 (−0.35, −0.07) [12.9%] **0.0026**	−0.20 (−0.33, −0.06) [12.2%] **0.0043**
AFI	−0.04 (−0.17, 0.08) [3.0%] 0.4946	−0.01 (−0.13, 0.12) [0.4%] 0.9312	−0.30 (−0.43, −0.17) [25.0%] <**0.0001**	0.04 (−0.09, 0.16) [−2.6%] 0.5511	−0.26 (−0.38, −0.13) [21.4%] **0.0001**	−0.30 (−0.42, −0.17) [24.6%] <**0.0001**
ALB	0.02 (−0.15, 0.19) [−1.4%] 0.8325	−0.01 (−0.17, 0.16) [0.4%] 0.9491	−0.35 (−0.52, −0.18) [37.8%] <**0.0001**	−0.02 (−0.19, 0.14) [1.9%] 0.7835	−0.37 (−0.54, −0.20) [39.7%] <**0.0001**	−0.34 (−0.51, −0.17) [37.2%] <**0.0001**
ALI	−0.01 (−0.13, 0.11) [1.3%] 0.8669	−0.04 (−0.15, 0.08) [4.8%] 0.5423	−0.27 (−0.39, −0.16) [52.9%] <**0.0001**	−0.03 (−0.14, 0.09) [3.4%] 0.6601	−0.26 (−0.38, −0.15) [51.0%] <**0.0001**	−0.24 (−0.36, −0.12) [46.0%] <**0.0001**
PFB	−0.04 (−0.15, 0.06) [2.3%] 0.4081	−0.09 (−0.19, 0.02) [4.7%] 0.1060	−0.15 (−0.25, −0.04) [8.3%] **0.0060**	−0.04 (−0.15, 0.06) [2.3%] 0.4299	−0.10 (−0.21, 0.00) [5.9%] 0.0544	−0.06 (−0.17, 0.04) [3.5%] 0.2452
PFI	−0.02 (−0.12, 0.08) [1.4%] 0.7055	−0.03 (−0.13, 0.07) [2.5%] 0.5099	−0.21 (−0.31, −0.11) [17.7%] <**0.0001**	−0.01 (−0.11, 0.09) [1.1%] 0.7799	−0.19 (−0.29, −0.09) [16.1%] **0.0003**	−0.17 (−0.27, −0.07) [14.9%] **0.0008**
PLB	−0.06 (−0.17, 0.05) [9.1%] 0.2545	0.00 (−0.10, 0.11) [−0.6%] 0.9342	−0.18 (−0.29, −0.07) [31.8%] **0.0011**	0.07 (−0.04, 0.17) [−8.8%] 0.2227	−0.12 (−0.23, −0.01) [20.9%] **0.0324**	−0.18 (−0.29, −0.08) [32.6%] **0.0008**
PLI	0.00 (−0.07, 0.07) [−0.3%] 0.9792	0.00 (−0.07, 0.07) [0.2%] 0.9840	−0.13 (−0.20, −0.05) [55.8%] **0.0007**	0.00 (−0.08, 0.07) [0.5%] 0.9633	−0.13 (−0.20, −0.05) [56.2%] **0.0008**	−0.13 (−0.20, −0.05) [55.4%] **0.0008**

Bolded *p*-values indicate statistical significance

*A* Anterior, *F* Facial, *B* Body, *I* Interproximal, *L* Lingual, *P* Posterior

^a^Difference is adjusted mean; a negative difference favours first named treatment

^b^Second named treatment taken as reference for percent difference calculation ((difference/reference) × 100%)

Fifteen subjects had a repeat plaque assessment and were included in the repeatability population. There was excellent agreement between first and second plaque assessments (weighted kappa = 0.927, 95% confidence interval (CI) = 0.913, 0.941).

### Safety

All 56 subjects were included in the safety population. No treatment-emergent AEs, including OST abnormalities, were reported with any of the dentifrices.

## Discussion

In this study, TPI score decreased from pre-brushing to post-brushing in all treatment groups, with statistically significantly greater reduction in plaque for dentifrices containing 67% (with or without herbs) or 62% NaHCO_3_ compared with a dentifrice containing 0% NaHCO_3_. These findings add to the current body of evidence that demonstrate that NaHCO_3_-containing dentifrices have greater plaque removal efficacy than those without NaHCO_3_.^4,6,14,15^ A recent 6-month study with a 67% NaHCO_3_ dentifrice found 11–15% decreases in TPI alongside differences in measures of gingivitis including visual elements, such as inflammation and gum bleeding on probing (almost 50% after 24 weeks).^16^ Interestingly, no differences were reported between dentifrices containing 67% and 62% NaHCO_3_ or between the 67% NaHCO_3_ dentifrices with or without herbs. While contrary to the earlier literature that suggested that it was the combination of herbs and NaHCO_3_ that contributed to anti-gingivitis effects,^7,8^ our findings support a more recent in vitro study that a toothpaste with the herbal ingredients traditionally included in some NaHCO_3_ dentifrices did not appear to have a significantly better action against the plaque biofilm when compared to one without herbal ingredients.^9^

A post hoc analysis to investigate specific areas of the dentition (the anterior or posterior aspects of the facial or lingual surfaces and interproximal [distal, medial] or body regions) found statistically significant differences in favour of the NaHCO_3_ dentifrices over the 0% NaHCO_3_ dentifrice for almost all areas of the mouth, with the greatest relative benefit in the anterior and posterior lingual interproximal areas where ~50% plaque was removed. This validates the use of the six site TPI as a measure of plaque levels as differences were found in hard-to-reach areas and percentage differences changed by region. This also supports the finding of other studies, including those over 6 months, that have indicated that brushing with NaHCO_3_ dentifrices has a plaque-reducing effect in more sheltered areas of the dentition^6,14,16^ and suggests that it is not only the mechanical action of brushing but also chemical action of NaHCO_3_ that contributes to lowered TPI scores. A recent in vitro study has indicated that the action of NaHCO_3_ against plaque biofilms may be related to its efficacy in disrupting the exopolysaccharide matrix structure.^10^

In conclusion, in this study plaque removal was significantly greater with 67% (with or without herbs) and 62% NaHCO_3_-containing dentifrices than a commercial 0% NaHCO_3_-containing dentifrice in a single, timed brushing clinical model. Further work in longitudinal studies is underway to examine whether the benefits of NaHCO_3_-containing dentifrices persist over time.
